# Complete Mitochondrial Genome of *Contracaecum* Sp. (Nematoda: Ascarididae) from Night Herons in China

**DOI:** 10.2478/jofnem-2022-0048

**Published:** 2022-10-26

**Authors:** Yuan-Ping Deng, Rong Li, Hui-Mei Wang, Guo-Hua Liu, Ya Tu

**Affiliations:** 1 Research Center for Parasites & Vectors, College of Veterinary Medicine, Hunan Agricultural University, Changsha, Hunan 410128, China; 2 Beijing Wildlife Rescue and Rehabilitation Center, Beijing 101300, China

**Keywords:** AT content, *Contracaecum*, *Contracaecum* sp, mitochondrial genome, mitogenome

## Abstract

*Contracaecum* species are zooparasitic anisakid nematodes and occur in gastrointestinal tracts of vertebrate/invertebrate animals, including humans, causing gastrointestinal pain, diarrhea, and increasingly severe vomiting. Although the complete mitochondrial (mt) genome (mitogenome) of *Contracaecum* sp. isolated from night herons in Beijing has been reported, the detailed information about this mt sequence is still puzzling. In the present study, we described the detailed characteristics across the complete mt DNA of *Contracaecum* sp., which includes 36 genes consisting of 12 protein genes, 22 transfer RNAs (tRNAs), 2 ribosomal RNAs (rRNAs), and 2 noncoding regions (NCRs), and all genes have the same orientation of transcription. The AT content in the complete mitogenome of *Contracaecum* sp. was 72.2%, and it was the least value (66.7%) in the *cox*1 gene but was the highest rate (84.1%) in NCRs. The highest nucleotide diversity (Pi) among the genus *Contracaecum* was *nad*4 (0.190) and the least was *cox*1 (0.125), which indicates that *nad*4 might have the potential ability as useful markers to detect cryptic species in the genus *Contracaecum* or subspecies. Based on the maximum likelihood (ML) and Bayesian inference (BI) computational algorithms within subfamilies Ascaridoidea and Heterakoidea, the results supported that *Contracaecum* sp. was a new species and the family Ascaridiidae was paraphyletic. The complete mitogenome sequence of *Contracaecum* sp. supported a clear recognition of *Contracaecum* species and provided the potential existence of cryptic species in the genus *Contracaecum*. Our findings would better contribute to the surveillance, molecular epidemiology, and control of *Contracaecum*.

*Contracaecum* species are nematodes that parasitize throughout the world, causing severe pathogenic influences on vertebrate and invertebrate animals, including humans (Shamsi, 2019). They have an indirect life cycle: first intermediate hosts involve a wide range of invertebrates, including cephalopods, copepods, and crustaceans; and second intermediate hosts are various fishes; and piscivorous birds are definitive hosts, causing severe diseases in birds like hemorrhages, necrosis, and severe ulcerative eosinophilic granulomas in intestinal tracts (Zhang et al., 2021). In Australia, the first anisakid nematode was detected in the human body coupled with gastrointestinal pain, diarrhea, and increasingly severe vomiting, although it was not identified at the species level (Shamsi and Butcher, 2011; Shamsi et al., 2019). Over the years, anisakidosis caused by *Contracaecum* was diagnosed mainly based on its morphological characteristics.

In the past decades, morphological features and molecular sequences of >100 *Contracaecum* species have been described (Shamsi et al., 2019; Zhang et al., 2021). However, it was challenging for nonexperts to distinguish and identify specific helminths based only on morphological features, especially for detecting cryptic species. Studies show that there are considerable differences between cryptic subspecies/species which are isolated from different hosts or geography (Shamsi et al., 2009; Mattiucci et al., 2014; Timi et al., 2014; Liu et al., 2016). The complete mitochondrial (mt) genome (mitogenome) has been evidenced as a useful molecular marker to identify and distinguish different/ similar species between related taxa, even cryptic species, especially for *Contracaecum* (Mohandas et al., 2014). However, only three *Contracaecum* species published complete mitogenome sequences, *C. ogmorhini*, *C. osculatum*, and *C. rudolphii*, which made it difficult to detect new/cryptic species within the genus *Contracaecum*. Although the complete mitogenome of *Contracaecum* sp., which was collected from black night herons from Beijing, China, has been published (GenBank no. MN892395), the published sequence was not characterized and detailed features were not recorded, which caused inconvenience to use it.

Therefore, in the present study, we aim to (i) reassemble and annotate the complete mitogenome of *Contracaecum* sp., which was isolated from black herons in Beijing, China, and describe detailed information of this sequence; (ii) based on uploaded annotated sequences of Ascaridoidea and Heterakoidea species, conduct phylogenetic analyses to verify Zhang et al. (2021) hypothesis; and (iii) provide more detailed molecular features and new useful markers for detect cryptic species within genus *Contracaecum* for successive studies.

## Materials and Methods

### Parasites and molecular identification

Helminth specimens were obtained from the digestive tracts of gray and night herons in Beijing Zoo, China. The species were washed with ultrapure water and physiological saline solution, fixed in 75% ethanol, and stored at -40°C. The specimens were preliminarily identified as *Contracaecum* based on hosts and primary characteristic morphology (Zhang et al., 2021). For additional examination of molecules, the total genomic DNA was extracted using a QIAampÒ DNA Micro Kit as per the manufacturer’s instructions. Based on polymerase chain reaction (PCR) amplification of partial *cox*1 (with primers JB3 – JB4.5) (Bowles et al., 1992; Bowles and McManus, 1994) and ITS (including ITS-1, 5.8S, and ITS-2) (with primers NC5 – NC2) (Newton et al., 1998; Chilton et al., 2001), the worms were recognized at the species level. The obtained ITS sequence was totally matched with published *Contracaecum* sp. (GenBank no. MW538933~36), and the partial *cox*1 sequence showed 99.7% identity with *Porrocaecum reticulatum* (GenBank no. MF113244).

### Sequencing, assembling, and annotation

The genomic DNA sample was fragmented to a size of 350 bp. The DNA libraries were sequenced using high-throughput sequencing (HTS) on an Illumina Hiseq 6000 platform (Novogene Co. Ltd., Tianjin, China), and 250-bp paired-end reads were generated. The raw data were obtained and recorded in FASTQ format. Then, the reads with low-quality bases (Phred quality <5) or uncertain reads with repetitive “N” bases were filtered to acquire clean data. The partial *cox*1 sequence was used as the initial reference to assemble complete mt sequence of *Contracaecum* sp. using Geneious Prime 2022.0.1 (Kearse et al., 2012). The assembly was operated with the following parameters: (i) minimum overlap within the range of 150 bp to 200 bp; (ii) minimum overlap identity among 98% to 100%; and (iii) maximum gap of 5 bp. The assembled mitogenome was verified by long PCR with designed primers (Table S1 and Fig. S1 in Supplementary Materials).

ORF Finder (https://www.ncbi.nlm.nih.gov/orffinder/) was used to identify the start/stop codons and boundaries of protein-coding genes (PCGs). Later, two ribosomal RNAs (rRNAs, *rrn*L and *rrn*S) were framed using Tandem repeats finder (Benson, 1999). The 12 protein genes were then further confirmed with previously published Ascarididae sequence (*Contracaecum osculatum*, GenBank no. JN786330). The tRNAscan-SE 2.0 (Chan et al., 2021) with a cutoff score of 1.0 and MITOs (Bernt et al., 2013) were applied to search 22 potential transfer RNAs (tRNAs).

### Nucleotide variation in mtDNA genomes among Contracaecum spp

Based on available mitogenome sequences of the genus *Contracaecum* in the NCBI, mt sequences were aligned using Clustal X1.83 to a single alignment dataset, including *C. osculatum*, *C. rudolphii*, *C. ogmorhini*, and *Contracaecum* sp. The nucleotide diversity of *Contracaecum* species was computed by DnaSP v5 using sliding windows (Librado and Rozas, 2009). The parameters of the sliding window were followed with 300-bp window length and a default 25-bp step site to calculate the nucleotide diversity (Pi or p). Each boundary of protein genes was identified due to mid-point position, and we then graphed nucleotide diversity for 12 protein genes from *Contracaecum*.

### Phylogenetic analyses

A total of 41 mitogenomes of species from families Ascaridoidea and Heterakoidea were applied to analyze phylogeny with outgroups *Enterobius vermicularis* (GenBank accession no. EU281143) and *Wellcomia siamensis* (GenBank accession no. NC_016129) (Table S2 in Supplementary Material). Each amino acid sequence was aligned using a MAFFT computational algorithm (Katoh et al., 2019). The aligned sequences were then concatenated to a single alignment dataset. The ambiguous gaps in the alignment were excluded by Gblocks 0.91b with default parameters “less stringent” (Dereeper et al., 2008). Computational algorithm maximum likelihood (ML) (Guindon et al., 2010) was conducted to perform a phylogenetic tree with the best model “JTT+I+G+F” screened by ProtTest 3.4.2 (Darriba et al., 2011) and applied 1,000 replicates. Bayesian analysis was operated with MrBayes 3.2 (Ronquist et al., 2012), and “GTR + F + G” was selected as the most suitable model by ModelFinder in IQTree v.2.1.3 (Kalyaanamoorthy et al., 2017). Four Markov chains were progressed with 1,000,000 MCMC generations, with sampling analysis tree each 100 generations. The residual trees were calculated with Bayesian posterior probabilities (BPP), burning first 250 trees.

## Results and Discussion

### Mitogenome organization and composition

**Figure 1:**
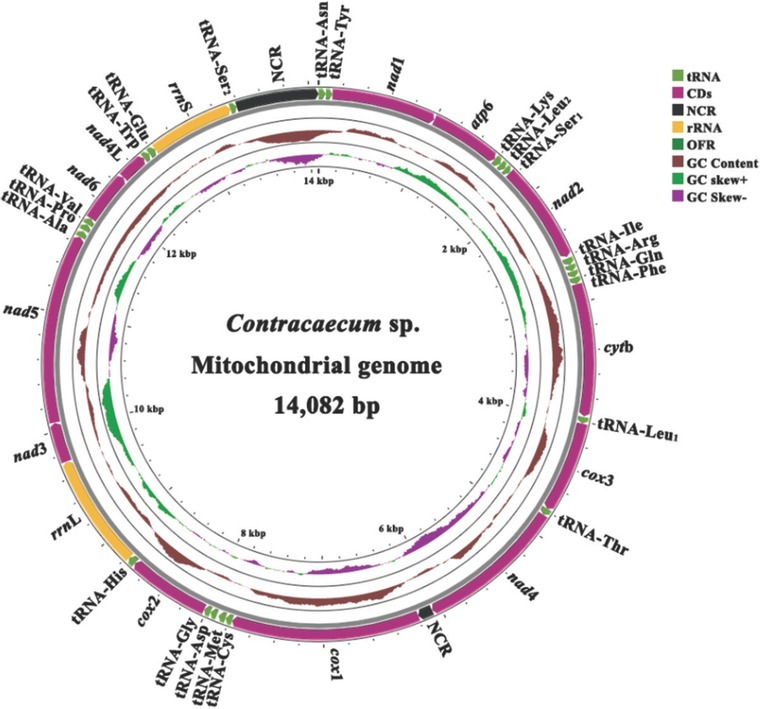
Organization of the complete mitochondrial genome sequence of *Contracaecum* sp. NCR, noncoding region.

The clean data of *Contracaecum* sp. are nearly 2 GB with a total of 8,677,194 × 2 clean reads for further assembling. The circular mt genome of *Contracaecum* sp. (GenBank accession: ON149889) assembled was 14,082 bp in size, shorter than that Zhang et al. (2021) published, with 12 PCGs, 22 tRNAs, 2 rRNAs, and 2 noncoding regions (NCRs) (Table 1 and Fig. 1). A total of 36 genes were transcribed in the forward direction and gene arrangement was recognized as the typical GA3 pattern, which is mostly observed in the worms (Liu et al., 2013). Consistent with previous reports, there was an obvious bias of A + T bases (71.2%). A total of 10 intergenic regions were found among the complete mt genome of *Contracaecum* sp. ranging from 1 bp to 16 bp (Table 1). One short NCR (122 bp) was located between *nad*4 and *cox*1, and one long NCR (691 bp) was placed in tRNA-Ser2 and tRNA-Asn. The values of AT skew were negative from -0.475 (*nad*6) to -0.111 (NCRs), and inversely, the values of GC-skew were positive with scope 0.226 (*nad*4) to 0.674 (*nad*3), suggesting Ts and Gs were more frequently used in the genome.

### Protein-coding genes

TTG was the most common initial codon in this study, followed by ATT. TTG was used as the start codon for nine genes (*cox*1-3, *cyt*b, *nad*1-4, and *nad*6) (Table 1). The rest three PCGs (*atp*6, *nad*4L, and *nad*5) used ATT as the initial codon. Generally, TAG and TAA were common stop codons in metazoans (Hu et al., 2004). In this study, TAA was the most frequent termination among *nad*6, *nad*4L, *nad*4, *cyt*b, and *nad*2. The genes *nad*1 and *nad*3 used TAG as their stop codon. The rest genes used incomplete stop codons T (*cox*1 and *cox*3) or TA (*atp*6, *cox*2 and *nad*5), respectively.

A total of 3,422 amino acids were translated by 12 PCGs. TTT (480) was the most common codon used in encoding Phe, followed by GTT (219, Val), TTG (216, Leu), and ATT (214, Ile). Leu (519) and Phe (499) were the most frequent amino acids, while Arg (34) was the least. There was a tendency of Gs and Ts in the same amino acid by comparing the relative synonymous codon usage (RSCU) (Table 2). The AT content of 12 protein genes ranged from 66.7% (*cox*1) to 78.9% (*nad*6) (Table 3). The values of AT skew ranged from –0.475 (nad6) to –0.373 (*cox*2), while the values of GC skew were 0.226 (*nad*4) to 0.674 (*nad*3), suggesting the bias of T and G bases.

### Transfer RNA genes, rRNA genes, and non-coding region

The length of 22 tRNAs ranged from 51 bp (tRNA-Ser1) to 65 bp (tRNA-His). The typical structure of tRNA consisted of one acceptor stem, a dihydrouridine loop (D-loop), an anticodon loop, TYC loop, and related arms fixing with them (Su et al., 2020). However, the TψC loop was always replaced by a TV replacement loop in nematodes. In our study, 16 of 20 tRNAs (excluding tRNA-Ser1 and tRNA-Ser2) lacked a TYC loop, replaced by several nucleotide residues, which compromised the TV replacement loop (Hu et al., 2004). The tRNA-His, tRNA-Ile, and tRNA-Met were observed in a relatively standard cloverleaf structure with a TYC loop, although the latter two (tRNA-Ile and tRNA-Met) lacked DHU stem. The tRNA-Ser1 and tRNA-Ser2 were similar to previous reports with one TYC-loop but lacked D-loop (Su et al., 2020).

**Table 1:** Organization of the complete mt genome of *Contracaecum* sp. from Beijing, China.

Gene/region	Strand	Positions	Size (bp)	Number of aa^a^	Ini/Ter codons	Anticodons	In
tRNA-Asn (N)	H	1–60	60			GTT	0
tRNA-Tyr (Y)	H	61–116	56			GTA	0
*nad*1	H	117–989	873	290	TTG/TAG		0
*atp*6	H	993–1,591	599	199	ATT/TA		+3
tRNA-Lys (K)	H	1,592–1,653	62			TTT	0
tRNA-Leu2 (L_2_)	H	1,654–1,708	55			TAA	0
tRNA-Ser1 (S_1_)	H	1,709–1,759	51			TCT	0
*nad*2	H	1,760–2,605	846	281	TTG/TAA		0
tRNA-Ile (I)	H	2,619–2,678	60			GAT	+13
tRNA-Arg (R)	H	2,679–2,732	54			GCG	0
tRNA-Gln (Q)	H	2,733–2,787	55			TTG	0
tRNA-Phe (F)	H	2,788–2,846	59			GAA	0
*Cyt*b	H	2,847–3,953	1,107	368	TTG/TAA		0
tRNA-Leu1 (L_1_)	H	3,961–4,017	57			TAG	+7
*cox*3	H	4,018–4,782	766	255	TTG/T		0
tRNA-Thr (T)	H	4,783–4,843	60			TGT	0
*nad*4	H	4,844–6,073	1,230	409	TTG/TAA		0
Intergenic region	H	6,074–6,195	122				0
*cox*1	H	6,196–7,771	1,576	525	TTG/T		0
tRNA-Cys (C)	H	7,772–7,829	58			GCA	0
tRNA-Met (M)	H	7,831–7,890	60			CAT	+1
tRNA-Asp (D)	H	7,907–7,963	57			GTC	+16
tRNA-Gly (G)	H	7,965–8,021	57			TCC	+1
*cox*2	H	8,022–8,713	692	230	TTG/TA		0
tRNA-His (H)	H	8,714–8,778	65			GTG	0
*rrn*L	H	8,779–9,737	959				0
*nad*3	H	9,738–10,073	336	111	TTG/TAG		0
*nad*5	H	10,077–11,659	1,583	527	ATT/TA		+3
tRNA-Ala (A)	H	11,660–11,716	57			TGC	0
tRNA-Pro (P)	H	11,724–11,780	57			TGG	+7
tRNA-Val (V)	H	11,781–11,837	57			TAC	0
*nad*6	H	11,838–12,272	435	144	TTG/TAA		0
*nad*4L	H	12,275–12,505	231	76	ATT/TAA		+2
tRNA-Trp (W)	H	12,506–12,563	58			TCA	0
tRNA-Glu (E)	H	12,565–12,624	60			TTC	+1
*rrn*S	H	12,625–13,335	711				0
tRNA-Ser2 (S_2_)	H	13,336–13,391	56			TGA	0
Noncoding region	H	13,392–14,082	691				0

a Inferred length of aa sequence of 13 PCGs. aa, amino acid; In, intergenic nucleotides; Ini/Ter codons, initiation and termination codons; PCGs, protein-coding genes; tRNA, transfer RNA

Ribosomal RNAs of *Contracaecum* sp. were fixed as a GA3 pattern. The *rrn*L was located between tRNA-His and *nad*3 with a size of 959 bp, and the rrnS gene was located between tRNA-Glu and tRNA-Ser2 with a size of 711 bp (Table 1). The content of A + T for *rrn*L and *rrn*S was 75.6% and 70.6%, respectively. There were two NCRs among the mt genome of *Contracaecum* sp. One short region was placed in *nad*4 and *cox*1 with a length of 122 bp, and the long region was situated between tRNA-Ser2 and tRNA-Asn with a length of 691 bp.

**Table 2:** Amino acid frequency of *Contracaecum* sp. mitochondrial PCGs.

Amino acid	Codon	Number	RSCU (%)	Amino acid	Codon	Number	RSCU (%)
Phe	TTT	480	1.92	Tyr	TAT	154	1.84
Phe	TTC	19	0.08	Tyr	TAC	13	0.16
Leu	TTA	199	2.3	Stop	TAA	5	1.43
Leu	TTG	216	2.5	Stop	TAG	2	0.57
Leu	CTT	76	0.88	His	CAT	54	1.86
Leu	CTC	2	0.02	His	CAC	4	0.14
Leu	CTA	10	0.12	Gln	CAA	20	0.98
Leu	CTG	16	0.18	Gln	CAG	21	1.02
Ile	ATT	214	1.92	Asn	AAT	100	1.79
Ile	ATC	9	0.08	Asn	AAC	12	0.21
Met	ATA	76	0.86	Lys	AAA	35	0.71
Met	ATG	101	1.14	Lys	AAG	63	1.29
Val	GTT	219	2.61	Asp	GAT	62	1.65
Val	GTC	13	0.16	Asp	GAC	13	0.35
Val	GTA	49	0.59	Glu	GAA	32	0.84
Val	GTG	54	0.64	Glu	GAG	44	1.16
Ser	TCT	139	3.08	Cys	TGT	53	1.96
Ser	TCC	6	0.13	Cys	TGC	1	0.04
Ser	TCA	14	0.31	Trp	TGA	21	0.57
Ser	TCG	5	0.11	Trp	TGG	53	1.43
Pro	CCT	66	3.11	Arg	CGT	33	3.88
Pro	CCC	7	0.33	Arg	CGC	1	0.12
Pro	CCA	9	0.42	Arg	CGA	0	0
Pro	CCG	3	0.14	Arg	CGG	0	0
Thr	ACT	89	3.24	Ser	AGT	121	2.68
Thr	ACC	6	0.22	Ser	AGC	2	0.04
Thr	ACA	9	0.33	Ser	AGA	36	0.8
Thr	ACG	6	0.22	Ser	AGG	38	0.84
Ala	GCT	72	2.5	Gly	GGT	112	2.22
Ala	GCC	24	0.83	Gly	GGC	21	0.42
Ala	GCA	11	0.38	Gly	GGA	23	0.46
Ala	GCG	8	0.28	Gly	GGG	46	0.91

Excluding abbreviated stop codons (TA and T). Stop = stop codon. PCGs, protein-coding genes; RSCU, relative synonymous codon usage.

**Table 3:** Nucleotide composition and skews of *Contracaecum* sp. mitochondrial genome.

		Nucleotide frequency (%)					
Gene	A	G	T	C	A + T (%)	AT-skew	GC-skew
*atp*6	22.0	22.0	49.1	6.9	71.1	-0.380	0.526
*cox*1	19.5	21.8	47.2	11.5	66.7	-0.416	0.307
*cox*2	21.2	22.1	46.5	10.1	67.7	-0.373	0.372
*cox*3	18.9	20.9	49.8	10.4	68.7	-0.449	0.333
*cyt*b	19.7	22.0	47.6	10.7	67.3	-0.415	0.343
*nad*1	19.5	20.5	50.5	9.5	70.0	-0.444	0.364
*nad*2	20.7	18.2	54.6	6.5	75.3	-0.451	0.474
*nad*3	20.0	21.4	54.4	4.2	74.4	-0.464	0.674
*nad*4	21.4	17.0	50.9	10.7	72.3	-0.408	0.226
*nad*4L	22.9	17.3	55.0	4.8	77.9	-0.411	0.569
*nad*5	21.2	18.8	51.9	8.1	73.1	-0.420	0.398
*nad*6	20.7	13.3	58.2	7.8	78.9	-0.475	0.261
*rrn*S	30.2	19.7	40.4	9.7	70.6	-0.143	0.340
*rrn*L	27.3	17.5	48.3	6.9	75.6	-0.277	0.436
22 tRNA	31.5	18.7	40.8	9.0	72.3	-0.129	0.352
NCR	37.4	10.3	46.7	5.6	84.1	-0.111	0.290
Total	23.5	19.0	48.7	8.9	72.2	-0.350	0.364

NCR, noncoding region.

### Nucleotide variation of genus Contracaecum

**Figure 2:**
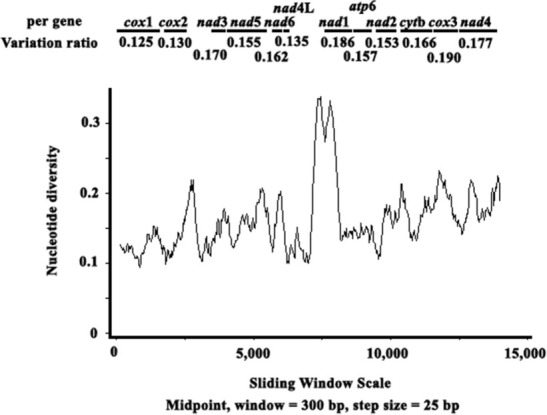
Sliding window analysis of the alignment of complete mtDNAs of available *Contracaecum* spp. The black line shows the value of nucleotide diversity Pi (π) in a sliding window analysis of window size 300 bp with step size 25 bp, and the value is inserted at its mid-point. Gene boundaries are indicated with a variation ratio per gene.

Based on aligned nucleotide sequences among species *C. osculatum*, *C. rudolphii*, *C. ogmorhini*, and *Contracaecum* sp., nucleotide diversities (Pi) were calculated based on the sliding window. The values of Pi ranged from 0.124 to 0.181 by analyzing a window of 300 bp and a default step of 25 bp (Fig. 2). The most variable genes were *cyt*b (0.178), *nad*2 (0.181), *nad*4 (0.179), and *nad*6 (0.172), and the most conserved genes were *cox*1 (0.124) and *cox*2 (0.130) in *Contracaecum* (Fig. 2). Protein genes *cox*1 and *cox*2 seemed to be the most stable genes in *Contracaecum* nematodes with the least variation, which could be used as molecular markers to identify species from *Contracaecum*. Results also supported that *nad*2 and *nad*4 could act as alternative markers among nematodes isolated from different distributions.

### Phylogenetic analyses

**Figure 3:**
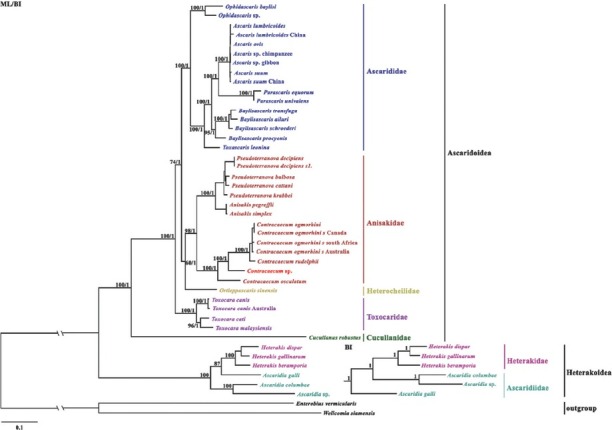
Phylogenetic relationships of *Contracaecum* spp. with species from Ascaridoidea and Heterakoidea. Analysis trees based on amino acid sequences of 12 protein genes by complete mitochondrial genome using BI and ML with *Enterobius vermicularis* and *Wellcomia siamensis* as outgroups. BI, Bayesian inference; ML, maximum likelihood.

The present phylogenetic trees were constructed based on the 12 PCGs of 41 available mt genome sequences from the superfamilies Ascaridoidea and Heterakoidea (Table S2 in Supplementary Material). Two phylogenetic trees, both Bayesian inference (BI) and ML, had similar topologies, excluding species within the superfamily Heterakoidea. The topologies of ML and BI phylogenetic trees were highly similar to those of previous studies (Liu et al., 2016; Zhang et al., 2021; Zhao et al., 2021). *Contracaecum* sp. formed a branch with *Contracaecum* nematodes, indicating a closer relationship within the genus with strong support (Fig. 3); however, the long distance between *Contracaecum* sp. and the other three *Contracaecum* species (*C. osculatum*, *C. rudolphii*, and *C. ogmorhini*) was longer than the branch distance within other anisakid nematodes, which further indicated *Contracaecum* sp. was a novel species and verified the hypothesis of Zhang et al. (2021) proposed. According to the structure of phylogenetic trees, results supported that the superfamilies Ascaridoidea and Heterakoidea were monophyletic and evidenced families, including Ascarididae, Anisakidae, Heterocheiidae, Toxocaridae, and Cucullanidae, were monophyletic, consistent with previous studies (Li et al., 2018; Zhao et al., 2021).

Within the superfamily Ascaridoidea, both BI and ML showed identical topologies. Among the family Ascarididae, the genera *Ascaris*, *Baylisascaris*, *Toxascaris*, and *Parascaris* had a closer relationship than *Ophidascaris*, which was similar to Zhou et al. (2021) reported. Based on morphological descriptions, the genus *Ophidascaris* was classified as a member of the superfamily Ascaridoidea (Pinto et al., 2010), and phylogenetic analyses suggested the genus *Ophidascaris* was more related to the family Ascaridae. However, the distance between the genus *Ophidascaris* and other Ascaridae genera was longer, suggesting there was systematic controversy in the Ascaridae. In the present study, the family Ascarididae was closely related to the family Anisakidae (Fig. 3), different from the previous study where the family Ascarididae was closely related to Toxocaridae (Zhou et al., 2021). In addition, results also supported the monophyly of all 5 families and all 11 genera within the superfamily Ascaridoidea with strong support (BPP = 1, Bf >70, Fig. 3), consistent with records (Liu et al., 2016; Zhao et al., 2018).

Liu et al. (2013) confirmed that *Ascaridia columbae* was more related to *Ascaridia* sp. than *A. galli*. The phylogenetic analyses in the present study also confirmed this. In ML analysis, the topology showed that *A. galli* was more related to *Heterakis* species with high statistical support (Bf = 87), in line with Liu et al. (2016) studied. However, BI analysis presented a totally different topology from that of ML tree. *A. galli* formed a distinct branch from genera *Heterakis* and *Ascaridia* with strong support (BPP = 1), hypothesizing *A. galli* might be another genus. Results also showed the family Heterakidae was a sister taxon to Ascaridiidae, and phylogenetic analyses (BI and ML) suggested the family Ascaridiidae might be paraphyly.

## Conclusion

In the present study, we annotated the complete mitogenome sequence of *Contracaecum* sp. isolated from night herons and described its characteristics. Based on available mitogenome sequences of *Contracaecum* species, we also calculated the nucleotide diversity, indicating *cox*1 and *cox*2 could be used as effective markers to distinguish and identify other *Contracaecum* species. Results also supported the hypothesis of Zhang et al. (2021) proposed that *Contracaecum* sp. was a novel species, and evidenced that families Heterakidae + Ascaridiidae were closely related and all genera and families (excluding genus *Ascaridia* and family Ascaridiidae) were monophyletic.

## Figures and Tables

**Figure S1:**
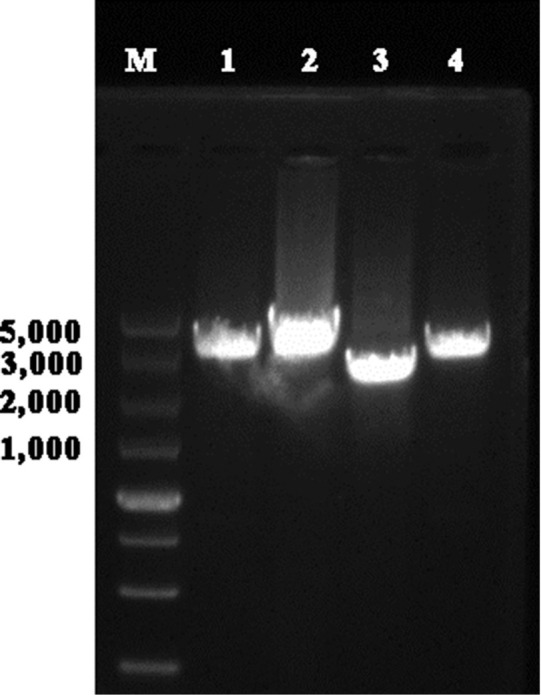
Polymerase chain reaction amplicons from the mitochondrial genome of *Contracaecum* sp. M: DL5,000 DNA marker; 1: Validation_01; 2: Validation_02; 3: Validation_03; 4:Validation_04.

**Table S1:** Primers used for assembly validation.

Name	Sequence (5^'^-3^'^)	Size (bp)
yeluF1	AGTTGTTGAAGAAGGAGCAGTT	
yeluR1	CTAAACATTGACCTAACCACCT	3,564 bp
yeluF2	AGGTGGTTAGGTCAATGTTTAG	
yeluR2	ACAGAGTAAACATCAGGGAAAT	3,900 bp
yeluF3	TTGGATTTCCCTGATGTTTACT	
yeluR3	CAAACTAAACATACTGCCAACA	2,816 bp
yeluF4	TTGGTCAACAAGATGGTCGTAA	
yeluR4	AACTGCTCCTTCTTCAACAACT	3,757 bp

**Table S2:** Mitochondrial genome sequences of nematodes of superfamily Ascaridoidea and Heterakoidea were sequenced completely before the present study and used for phylogenetic analysis.

Superfamily	Family	Species	Size (bp)	GenBank accession No.
Ascaridoidea	Anisakidae	*Anisakis pegreffii*	14,002	NC_034329
		*Anisakis simplex*	13,899	MK820679
		*Contracaecum ogmorhini Canada*	14,010	KU558727
		*Contracaecum ogmorhini Australia*	14,019	KU558725
		*Contracaecum ogmorhini South Africa*	14,012	KU558726
		*Contracaecum osculatum*	13,823	NC_024037
		*Contracaecum rudolphii*	14,022	NC_014870
		*Pseudoterranova decipiens*	13,962	NC_031645
		*Pseudoterranova decipiens s.l*.	13,965	KU558722
		*Pseudoterranova krabbei*	13,948	NC_031646
		*Pseudoterranova bulbosa*	13,957	KU558720
		*Pseudoterranova cattani*	13,950	KU558721
	Ascarididae	*Ascaris lumbricoides*	14,281	NC_016198
		*Ascaris lumbricoides China*	14,303	HQ704900
		*Ascaris ovis*	14,288	NC_036666
		*Ascaris suum*	14,284	NC_001327
		*Ascaris suum China*	14,311	HQ704901
		*Ascaris* sp. *Chimpanzee*	14,268	KC839986
		*Ascaris* sp. *gibbon*	14,274	KC839987
		*Baylisascaris ailuri*	14,657	HQ671080
		*Baylisascaris procyonis*	14,781	NC_016200
		*Baylisascaris schroederi*	14,778	NC_015927
		*Baylisascaris transfuga*	14,898	NC_015924
		*Ophidascaris baylisi*	14,784	MW880927
		*Ophidascaris* sp.	14,660	MK106624
		*Parascaris equorum*	13,899	NC_036427
		*Parascaris univalens*	13,920	NC_024884
		*Toxascaris leonina*	14,310	NC_023504
	Heterocheilidae	*Ortleppascaris sinensis*	13,828	NC_036669
	Toxocaridae	*Toxocara canis*	14,322	NC_010690
		*Toxocara canis Australia*	14,163	EU730761
		*Toxocara cati*	14,029	NC_010773
		*Toxocara malaysiensis*	14,266	NC_010527
	Cucullanidae	*Cucullanus robustus*	13,972	NC_016128
Heterakoidea	Ascaridiidae	*Ascaridia columba*e	13,931	NC_021643
		*Ascaridia galli*	13,977	NC_021642
		*Ascaridia sp*.	13,862	JX624730
	Heterakidae	*Heterakis beramporia*	14,012	NC_029838
		*Heterakis dispar*	13,995	NC_042411
		*Heterakis gallinarum*	13,973	NC_029839
Oxyuroidea	Oxyuridae	*Enterobius vermicularis*	14,010	EU281143
		*Wellcomia siamensis*	14,128	NC_016129
